# Genetic Characterization of *Legionella pneumophila* Isolated from a Common Watershed in Comunidad Valenciana, Spain

**DOI:** 10.1371/journal.pone.0061564

**Published:** 2013-04-25

**Authors:** Leonor Sánchez-Busó, Mireia Coscollá, Marta Pinto-Carbó, Vicente Catalán, Fernando González-Candelas

**Affiliations:** 1 Genomics and Health Joint Unit CSISP (FISABIO)-University of Valencia/Cavanilles Institute, Valencia, Spain; 2 CIBER Epidemiology and Public Health, Valencia, Spain; 3 Tuberculosis Research Unit, Swiss Tropical and Public Health Institute, Basel, Switzerland; 4 Labaqua, S.A. Alicante, Spain; Institut de Biologia Evolutiva - Universitat Pompeu Fabra, Spain

## Abstract

*Legionella pneumophila* infects humans to produce legionellosis and Pontiac fever only from environmental sources. In order to establish control measures and study the sources of outbreaks it is essential to know extent and distribution of strain variants of this bacterium in the environment. Sporadic and outbreak-related cases of legionellosis have been historically frequent in the Comunidad Valenciana region (CV, Spain), with a high prevalence in its Southeastern-most part (BV). Environmental investigations for the detection of *Legionella pneumophila* are performed in this area routinely. We present a population genetics study of 87 *L. pneumophila* strains isolated in 13 different localities of the BV area irrigated from the same watershed and compare them to a dataset of 46 strains isolated in different points of the whole CV. Our goal was to compare environmental genetic variation at two different geographic scales, at county and regional levels. Genetic diversity, recombination and population structure were analyzed with Sequence-Based Typing data and three intergenic regions. The results obtained reveal a low, but detectable, level of genetic differentiation between both datasets, mainly, but not only, attributed to the occurrence of unusual variants of the *neuA* locus present in the BV populations. This differentiation is still detectable when the 10 loci considered are analyzed independently, despite the relatively high incidence of the most common genetic variant in this species, sequence type 1 (ST-1). However, when the genetic data are considered without their associated geographic information, four major groups could be inferred at the genetic level which did not show any correlation with sampling locations. The overall results indicate that the population structure of these environmental samples results from the joint action of a global, widespread ST-1 along with genetic differentiation at shorter geographic distances, which in this case are related to the common watershed for the BV localities.

## Introduction

*Legionella pneumophila* is a Gram-negative bacterium commonly found in superficial-water ecosystems and in association with microbial biofilms [1]. From there, it is capable of colonizing urban and industrial water-supply systems, spreading into the environment through aerosols and causing infection when inhaled by susceptible persons.

*L. pneumophila* was first reported as a pathogen after an acute pneumonia outbreak in Philadelphia (USA) during a convention of the American Legion in 1976 [2]. Since then, numerous community- and travel-associated outbreaks of *Legionella*-associated cases have been reported. The most severe form of pneumonia caused by *Legionella* infection is known as Legionnaires‘ disease but there is also a milder, flu-like form known as Pontiac fever [3].

Although other species of *Legionella* are capable of producing infection, *L. pneumophila* serogroup 1 is responsible for about 84% of sporadic cases and outbreaks of legionellosis in the world and 95% in Europe [4]. These bacteria can multiply intracellularly in amoebas and other ciliate hosts [5]. Moreover, the pathogenesis of *Legionella* is comparable between amoebas and human macrophages [1]; in fact, these bacteria are able to enter their hosts by both traditional and coiling phagocytosis [6].

The currently accepted typing scheme for *L. pneumophila* is known as Sequence-Based Typing (SBT) [7], [8], a variant of the Multilocus Sequence Typing (MLST) method [9]. In the case of *L. pneumophila*, the SBT scheme is based on PCR amplification and sequencing of 7 loci, including two housekeeping genes (*asd*, *neuA*) and five genes associated with virulence (*fliC*, *pilE*, *mip*, *proA*, *mompS*). But these loci only provide genetic information on approximately 3 kb of the analyzed strains, in comparison to the whole *L. pneumophila* genome (approximately 3.5 Mb) [10]–[14]. In order to increase the level of resolution for epidemiological studies, Coscollá and González-Candelas [15] studied 13 intergenic regions of the bacterial genome. In fact, the combination of only three of these markers provided an index of discrimination (ID) of 0.88, exactly the same as the discriminating ability of the six genes established by EWGLI [16], which increased when combined with these intergenic regions.

Our research group has reported previously about the genetic variability of *L. pneumophila* strains distributed all around the Comunidad Valenciana (CV) region (Spain) isolated from different environments [15], [17]. This region is located along the East of Spain, in the Mediterranean coast, with a surface of 23,255 km^2^ and a population of about 5,000,000 inhabitants. Legionellosis outbreaks and cases have been frequent in this area, with the first documented outbreak having occurred in a hotel in 1973 [18], [19]. The main interest of this work resides on the characterization of the genetic variability, recombination and population structure of *L. pneumophila* strains isolated from a specific water distribution network, fed from the same watershed, in a localized area within the Comunidad Valenciana. This is a relatively small territory, with 1,000 km^2^ and 400,000 inhabitants located at the South of the CV (Alicante province), hereafter denoted as BV. We were interested in gaining insight on the distribution of genetic variation in this species at local geographic scales and its relation to those in related localities, in this case connected by the same major watershed.

## Materials and Methods

### Samples

A total of 133 environmental samples were included in this study. Of those, 87 were collected from 1998 to 2006 during regular surveillance for *Legionella* in different points of the water distribution network that supplies 13 populations in the Alicante province (Comunidad Valenciana, Spain) from the same watershed. These locations are denoted as BV. Sampling points were always pre-defined places of the water system where routine water quality control monitoring was usually performed and consisted in small and closed installations in the street for collecting representative water samples, avoiding cross-contamination. Data from 46 additional isolates were retrieved from a previous work by our group [17], and consisted of *L. pneumophila* environmental strains sampled around other localities of the Comunidad Valenciana (denoted as CV). Samples were obtained with permission from the Environmental Health Service, Conselleria de Sanidad, the authority in charge of environmental surveillance for water-borne pathogens across the Comunidad Valenciana, the Spanish region studied in this work. No clinical or human origin samples were used in this study and no animals were used in it.

Bacterial colonies from pure cultures were suspended in 200 µL of 20% Chelex 100 resin (Bio-Rad Laboratories, Richmond, CA). DNA was then extracted by three freeze-thaw cycles (4°C for 5 min and 99°C for 5 min), and cellular debris was removed by pelleting at maximum speed for 1 min. The quantity of genomic DNA and its purity were measured by spectrophotometry at 260 nm in triplicates using the A260/A280 ratio with NanoDrop™ 1000 (Thermo Scientific). Purified DNA was stored at −20°C until used.

### PCR Amplification and Product Purification

The seven regions of the *L. pneumophila* genome used for typing (*fliC*, *pilE*, *asd*, *mip*, *mompS*, *proA* and *neuA*) [8], [16] and three intergenic regions (L2, L6 and L14, [15]) were amplified by standard PCR.

Amplification mixtures contained 1X standard reaction buffer with 2 mM MgCl_2_ (Biotools), 1 U DNA polymerase (Biotools), 100 µM of each primer, 50 ng of sample DNA and ultrapure water until a final volume of 50 µl. The oligonucleotides used for the SBT scheme gene amplification were described by Gaia et al (2005), except those for *neuA*, which were designed by our group to improve amplification results (neuAB_F: ACCGATAGTAAACAAATAGC, neuAB_R: TTCTGTTAGAGCCCAATCGA, optimal melting temperature 56°C; Coscollá *et al*., unpublished), although other combination of primers were published in Farhat *et al*. [20].

The amplification program consisted of a 2 min denaturation step at 94°C, 35 cycles of denaturation (30 s at 94°C), annealing (30 s at the optimal annealing temperature for each pair of primers [15], [16] and extension (30 s at 72°C). Finally, the reaction was subjected to a final step at 72°C for 8 min.

PCR products were then purified using NucleoFast® 96 PCR Plates (Macherey-Nagel) following the centrifugation protocol, eluted in 50 µl of ultrapure water and finally stored at −20°C until sequencing.

### DNA Sequencing

Purified PCR products were subjected to Sanger sequencing using BigDye™ Terminator v3.0 Ready Reaction Cycle Sequencing Kit (Applied Biosystems) and analyzed in an ABI PRISM 3700 sequencer (Applied Biosystems, Foster City, CA). The program consisted of 60 cycles of 10 min at 94°C, 5 s at 50°C and 4 min at 60°C. The oligonucleotides used for sequencing were the same used in the amplification reaction except for *mompS*. In this case, an inner reverse primer was applied, as previously described [16]. Chromatograms were processed by *gap4* and *pregap4*, from the Staden package [21], to obtain a consensus sequence for each locus.

Newly determined sequences are publicly available in GenBank with accession numbers KC409659-KC410574.

### Sequence Analysis

Two concatenates of sequences were prepared, one with the six loci of the initial SBT scheme (without *neuA* and the three intergenic regions) [16] and the other with all ten loci. The concatenation was made using BioEdit (available from http://www.mbio.ncsu.edu/BioEdit/bioedit.html) according to their relative position in the *L. pneumophila* str. Philadelphia genome. Sequences were aligned with Muscle [22], [23], implemented in MEGA v5.0 [24].

The best substitution model for both concatenates was assessed with jModelTest [25]. The application of the Akaike Information Criterion (AIC) [26] resulted in the selection of GTR+Γ as the best model for these data, and this was used for phylogenetic reconstruction following the maximum likelihood (ML) method implemented in RAxML v7.2.8 [27].

### Genetic Variability

Genetic variability was analyzed with DnaSP v5.10.01 [28]. The studied parameters were the number of polymorphic sites (S), haplotype diversity (Hd) [29], nucleotide diversity (π) [29], average number of pairwise differences (k) [30], and population mutation rate per site (θ) [31].

### Topological Congruence

We investigated the topological congruence between the ML trees of each DNA fragment analyzed independently, and also with the trees resulting from two concatenates: 9 (without *neuA*) and 10 loci. As different methods for testing topology congruence have distinct drawbacks, potentially resulting in biased results, three different tests were performed. The Shimodaira-Hasegawa (SH, [32]) test is dependent on the best tree to be included among those considered in the test and behaves conservatively as the number of input trees increases. The Expected-Likelihood Weight (ELW) test [33] is independent of the true best tree, but it needs long sequence datasets to correct for possible model miss-specification. Finally, the Approximately Unbiased (AU) test [34] is based on a multiscale bootstrap to correct for selection bias, although, if many of the candidate trees are almost equally well supported, the best true tree might be missed due to an over-confidence in the wrong ones. In order to assess the potential rejection of any of the 12 tested topologies by each of the corresponding datasets, the first two tests were performed using TREE-PUZZLE v5.2 [35] and the third one with CONSEL [36].

### Recombination

RDP3 [37] was used to test for possible intergenic and intragenic recombination events in this dataset by applying seven detection methods: RDP [38], GENECONV [39], Bootscan/Recscan [40], MaxChi [41], Chimaera [42], SiScan [43] and 3Seq [44]. The circularity of the *Legionella* genome was taken into account for these tests. The significance level was established at p<0.05 and Bonferroni’s correction for multiple comparisons was applied. Only recombination events detected by at least two of the methods were considered.

As RDP3 detects potential breakpoints in the alignment, in order to confirm the distinct phylogenetic history of the regions involved in recombination events and their flanking fragments, the 10-loci alignment was split in two parts and ML trees were inferred for each one. One portion of the alignment included the putative recombinant region detected by RDP3 while the other included the concatenate of the corresponding flanking regions. Subsequently, these topologies were used for testing their reciprocal congruence with the corresponding alignments using the SH [32] and ELW tests [33] with TREE-PUZZLE v5.2 [35].

### Population Structure

Structure v2.3 [45] was applied for inferring population structure using haplotype data with no prior information. The potential number of populations (K) was assessed 10 times from K = 2 to K = 10 using the linkage model [46] with a burn-in of 20,000 generations and 100,000 MCMC iterations. The results were processed with Structure Harvester online (http://taylor0.biology.ucla.edu/struct_harvest/) and subsequently with CLUMPP v1.1.2 [47] to obtain a consensus result of the 10 replicates for each K value, which was graphically represented with Distruct [48]. The optimal value of K was assessed using Evanno’s method, which is based on the second-order rate of change of the log probability of the data between consecutive values of K [49].

Moreover, population structure was further studied by estimating variance components and F-statistics through an Analysis of Molecular Variance (AMOVA) [50], as implemented in Arlequin v3.5 (available from http://cmpg.unibe.ch/software/arlequin3). This analysis provides the percentage of genetic variation within and among populations (fixation index, F_ST_), which represents the correlation of random haplotypes within populations, relative to that of random pairs drawn from the whole species [50]. The statistical significance was non-parametrically tested using 10,000 random permutations.

### Neutrality Tests

We tested for deviations of neutrality in each of the 10 loci using Tajima’s D, Fu & Li’s D* and F* and Fu’s Fs tests as implemented in DnaSP v.5.10.01 [28]. Statistical significance was evaluated from 1,000 coalescent simulations and the false discovery rate (FDR) [51], [52] method was applied to correct for multiple comparisons (α = 0.025).

## Results

### Sequence Typing

The allelic profile of the 87 isolates from BV was obtained by sequencing the seven loci in the EWGLI typing scheme. For the 46 strains from CV, the *neuA* locus was sequenced, thus allowing the assignment of the corresponding sequence type (ST) from the information in the EWGLI database. Four *neuA* alleles in BV samples were widely divergent from those traditionally included in the EWGLI database, but have recently been described as a different group of *neuA* variants [20] (Table S1 in File S1) denoted as *neuAh*.

The 133 isolates corresponded to 30 different STs (Table S1 in File S1). Most of these (22 STs from 28 isolates) were exclusive to one single locality, and only three (ST-1, ST-777, and ST-1356) were present in more than 3 locations (Table S2 in File S1). The most frequent variant was ST-1, which corresponds to strain Paris [10], present in 63 isolates from 16 different localities. In fact, this ST was found in all but two sampling locations (CV-5 and BV-14) where only 4 and 1 samples had been taken, respectively. ST-1356 was present in 6 locations from BV, with a total of 21 isolates, whereas ST-777 was found in 4 localities, one from CV and three from BV, for a total of 6 isolates. Three additional STs (ST-48, ST-719, and ST-856) were shared by locations from the CV and BV.

### Genetic Variability

The main genetic variability parameters estimated from our data are shown in Table 1. These data allowed the comparison between the genetic variability of 87 *L. pneumophila* strains from BV and the 46 isolates from the rest of the CV. The 67 bp non-coding fragment at the beginning of the *pilE* region was analyzed separately. Also, the presence in BV of four divergent alleles in the *neuA* gene (see above) led us to split this locus into two groups for comparison purposes.

**Table 1:** Genetic variability by locus of the 133 isolates included in the study.

	n		m		h		Hd		π			S		θ (no recomb)		k (no recomb)		Syn		Non-Syn		dN/dS	
	BV	CV	BV	CV	BV	CV	BV	CV		BV	CV	BV	CV	BV	CV	BV	CV	BV	CV	BV	CV	BV	CV
**L14**	87	46	453	453	12	12	0.5850 (0.0430)	0.7950 (0.0500)		0.0421 (0.0025)	0.0478 (0.0030)	53	54	0.0239 (0.0067(	0.0278 (0.0086)	18.5540 (8.3037)	21.1070 (9.4859)	–	–	–	–	–	–
***proA***	87	46	440	440	4	7	0.5410 (0.0420)	0.6920 (0.0630)		0.0127 (0.0008)	0.0116 (0.0019)	13	19	0.0059 (0.0021)	0.0098 (0.0035)	5.5960 (2.6941)	5.1010 (2.5197)	13	18	0	1	0.0000	0.0050
***pilE*** **NC**	87	46	67	67	3	4	0.1110 (0.0460)	0.5610 (0.0410)		0.0027 (0.0012)	0.0165 (0.0013)	3	4	0.0089 (0.0054)	0.0136 (0.0075)	0.1800 (0.2366)	1.1020 (0.7355)	–	–	–	–	–	–
***pilE***	87	46	346	346	6	6	0.5570 (0.0390)	0.6930 (0.0410)		0.0097 (0.0013)	0.0224 (0.0009)	20	21	0.0115 (0.0038)	0.0138 (0.0048)	3.3560 (1.7378)	7.7650 (3.6831)	18	18	2	3	0.0580	0.0170
**L2**	87	46	481	481	7	10	0.5430 (0.0360)	0.8190 (0.0360)		0.0248 (0.0016)	0.0285 (0.0030)	46	49	0.0196 (0.0056)	0.0240 (0.0074)	11.5430 (5.2822)	13.2520 (6.0716)	–	–	–	–	–	–
***neuA+neuAh***	87	46	476	476	11	8	0.5600 (0.0500)	0.7480 (0.0480)		0.1433 (0.0150)	0.0105 (0.0004)	180	14	0.0756 (0.0194)	0.0067 (0.0025)	67.7820 (29.5004)	4.9810 (2.4668)	n.a.	10	n.a.	4	0.3030	0.1370
***neuAh***	24	46	476	–	4	–	0.2390 (0.1130)	–		0.0051 (0.0030)	–	22	–	0.0125 (0.0047)	–	2.4200 (1.3601)	–	19	–	3	–	0.0450	–
***neuA***	63	46	476	–	7	–	0.2650 (0.0730)	–		0.0045 (0.0013)	–	24	–	0.0107 (0.0035)	–	2.1230 (1.1979)	–	18	–	6	–	0.1320	–
***mip***	87	46	498	498	12	11	0.5850 (0.0430)	0.8440 (0.0400)		0.0047 (0.0009)	0.0099 (0.0011)	25	20	0.0100 (0.0031)	0.0091 (0.0032)	2.3340 (1.2876)	4.9250 (2.4423)	23	18	2	2	0.0110	0.0280
***fliC***	87	46	200	200	5	5	0.5420 (0.0360)	0.7430 (0.0380)		0.0174 (0.0010)	0.0180 (0.0014)	10	12	0.0099 (0.0039)	0.0137 (0.0054)	3.4860 (1.7944)	3.5970 (1.8590)	10	9	0	3	0.0000	0.0260
**L6**	87	46	407	407	7	8	0.5440 (0.0400)	0.7890 (0.0440)		0.0085 (0.0008)	0.0186 (0.0018)	22	29	0.0109 (0.0035)	0.0162 (0.0054)	3.4110 (1.7621)	7.5680 (3.5972)	–	–	–	–	–	–
***asd***	87	46	501	501	7	8	0.5290 (0.0360)	0.7490 (0.0530)		0.0070 (0.0004)	0.0089 (0.0005)	11	12	0.0044 (0.0017)	0.0055 (0.0021)	3.5070 (1.8036)	4.4680 (2.2419)	11	11	0	1	0.0000	0.0090
***mompS***	87	46	509	509	9	8	0.5740 (0.0350)	0.7760 (0.0450)		0.0078 (0.0013)	0.0130 (0.0027)	41	37	0.0161 (0.0047)	0.0166 (0.0053)	3.9490 (1.996)7	6.5520 (3.1537)	n.a.	n.a.	n.a.	n.a.	0.0650	0.0940

Both groups of *neuA* alleles found in BV and the 67 bp non-coding fragment of *pilE* (*pilE*NC) are analyzed separately. BV and CV account for the two datasets under study. Standard deviations are given in parentheses. **n:** Number of sequences. **m:** Sequence length. **h:** Number of haplotypes. **Hd:** Haplotype diversity. **π:** Nucleotide diversity. **S**: number of polymorphic sites. **k:** Number of pairwise differences. **θ:** Expected heterozygosity per site from S. **Syn:** Number of synonymous changes. **Non-Syn:** Number of non-synonymous changes. **ω:** dN/dS ratio. **n.a.:** Not available.

Haplotype diversity (Hd) correlates positively with the number of sequences in a sample. However, the estimates of Hd for the CV sample (n = 46) were higher than those in BV (n = 87) in all loci considered. A similar result was obtained for most comparisons between the two samples for the remaining genetic variability parameters in which the CV samples presented higher values than those from BV (Table 1). The only exceptions corresponded to the number of pairwise differences (k) and nucleotide diversity (π) in *proA* and the number of polymorphic sites (S) in *mip* and *mompS*. In *neuA*, the comparison between the two groups considering only the non-*neuAh* alleles yielded similar results to those previously reported, with only a few more polymorphic sites and mutations in the BV than in the CV samples.

In agreement with previous results [15], intergenic regions L2 and L14 were more diverse than the protein-coding loci. However, the intergenic region L6 presented a low diversity, especially in BV, comparable to that of coding fragments such as *fliC* or *mompS*, and even lower than the coding region of *pilE*. The genetic variability estimates in the two types of *neuA* alleles were similar and were among the lowest for all the loci considered. Only the non-coding portion of *pilE* presented lower estimates of nucleotide diversity, likely resulting from its small size (67 nt).

### Topological Congruence and Recombination Testing

Phylogenetic trees were constructed separately for each locus and for the concatenated alignment of all the loci. ELW and AU phylogenetic congruence tests between the 12 alignments and all the topologies resulted in the complete rejection of the null hypothesis for the topologies not directly derived from each alignment (Table S3 in File S1). In consequence, these results pointed to the independence of every DNA fragment analyzed from each other, an indication of the potential participation of these regions in intergenic recombination events.

**Figure 1:**
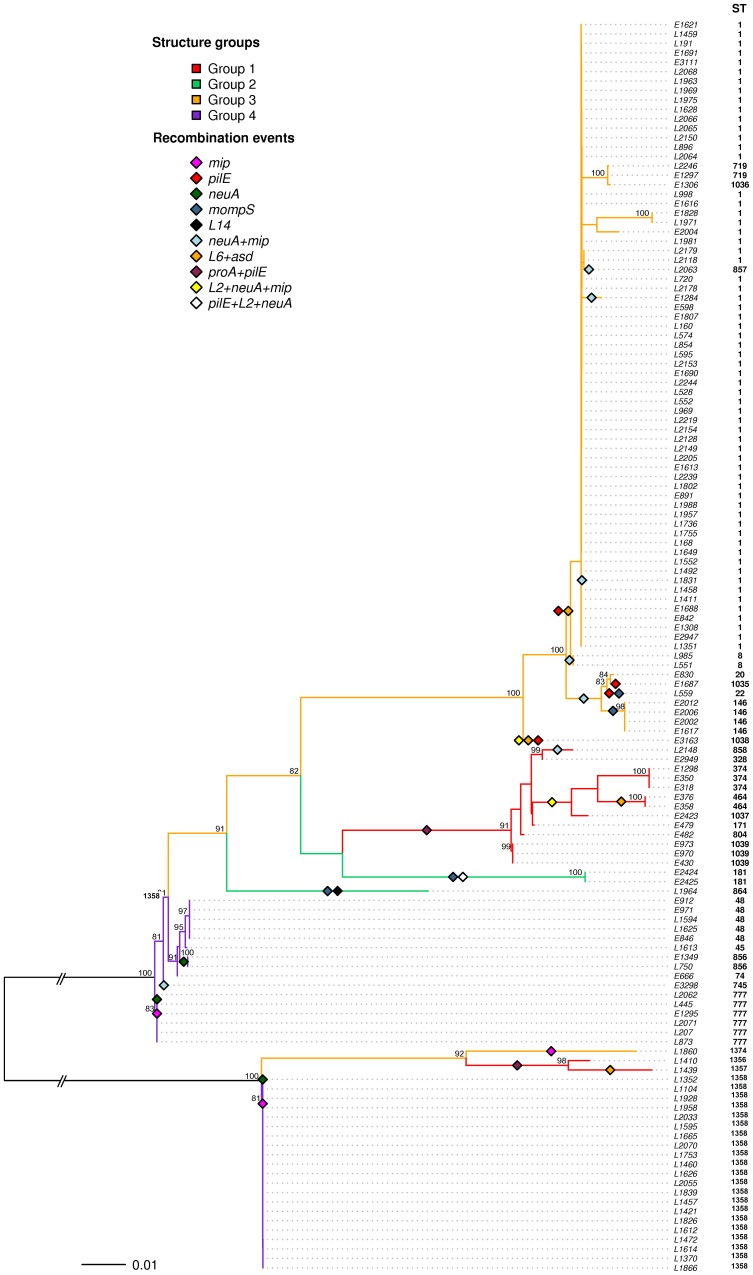
Maximum likelihood phylogenetic reconstruction of the 10-loci concatenate using partitioned data with RAxML. Colored clades represent the four groups detected by Structure (G1 in red, G2 in green, G3 in orange and G4 in purple). Sequence types (ST) of each sample are represented next to the tips of the tree. Colored rhombuses on the branches represent recombination events detected by RDP3. Bootstrap support values higher than 80% are shown.

The possibility that recombination might explain the observed lack of congruence was tested using RDP3 with the 36 haplotypes resulting from the concatenate alignment of the 10 loci from the 133 *L. pneumophila* strains (Figure 1; Table S4 in File S1). A total of 31 recombination events were detected, most of them by six or seven of the recombination detection methods implemented in the program, and 33 haplotypes showed from one to three recombination events. A single event involving the *pilE* region was found in the clade of the phylogram containing the isolates of ST-1, ST-8, ST-719, ST-857, ST-1036 and ST-1038 (Figure 1). A joint event including *proA* and *pilE* was also found in ten of the haplotypes and another one involving *neuA* and *mip* was detected in 13 haplotypes. The mapping of these events onto the ML phylogenetic tree (Figure 1) indicates that several *neuA*+*mip* events might have happened independently in the genealogical history of these isolates. However, recombination events in *neuA* and *mip* were also detected independently in three haplotypes (ST-1358, ST-777 and ST-856) and some of the *neuA+mip* events also involved locus L2, as shown in Figure 1. Another frequent recombination event included L6+*asd*, which might have happened in the ancestral node of the clade containing isolates ST-1 and ST-8, and also independently in the isolates E3163 and L1439. Locus *mompS* was also detected as being involved in several independent intergenic recombination events but locus L14 was detected as recombinant by four methods only in isolate L1964.

Apart from the statistical significance of each event given by the different methods implemented in RDP3, the alignment and the ML topology of the fragment within the detected breakpoints were compared with the alignments and ML topologies of the flanking regions for each recombination event. All reciprocal comparisons resulted in each alignment significantly rejecting the topology given by the other alignment, thus confirming the real existence of incongruent genealogical histories for the genomic stretches involved in all the inferred recombination events.

### Population Structure

In order to investigate the extent and distribution of genetic differentiation among the samples studied, we considered two datasets, one including the *L. pneumophila* strains isolated from the same watershed (BV), and the other with isolates from different locations in the Comunidad Valenciana (CV). The phylogenetic reconstructions for each locus and the concatenated alignment failed to group variants by sampling location except for the *neuA* locus, for which one well-defined cluster contained all newly described *neuA* alleles from BV (Figure 1). To gain further insight on the geographic structure of these 133 isolates at the genetic level, we used the Bayesian clustering method implemented in Structure, and applied it to both the 9-loci (Figure S1) and 10-loci concatenates (Figure 1) separately using the linkage model. This method assumes genomes admixture and also the possibility of linked loci coming from the same population. The objective of this double approach was to take into account the potential effect of the newly described *neuA* alleles in the estimation of the global genetic structure.

**Figure 2:**
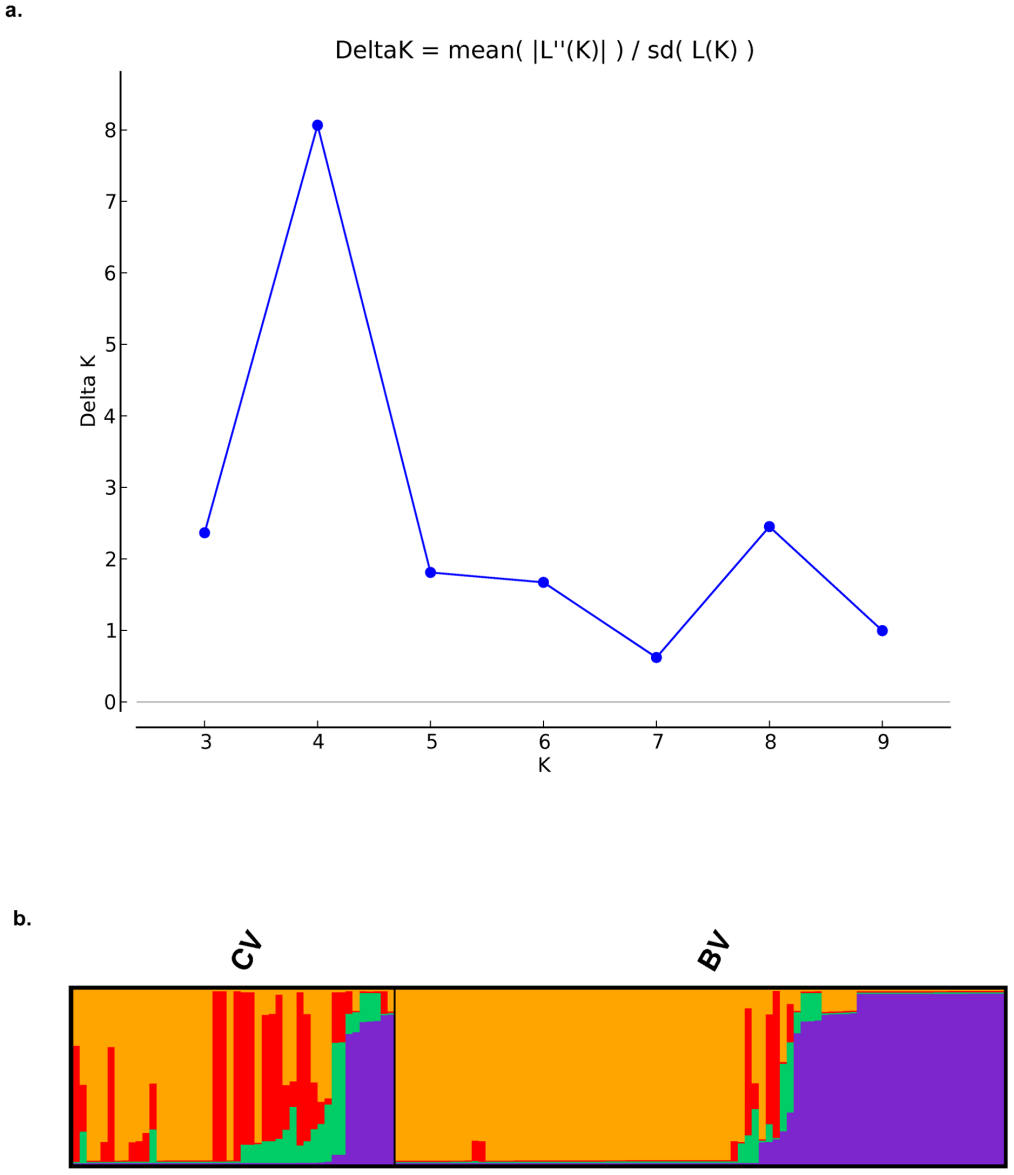
Summary of population assignment analyses using Structure. (a) Delta K values calculated by Evanno’s method detecting K = 4 groups as the most genetically probable within the 10-loci data by Structure Harvester Online. (b) Bar plot representing the probabilities of assignation of the isolates included in the study to each genetic group detected by Structure within CV and BV.

From the different number of populations which were assumed *a priori* (from K = 2 to K = 10), both concatenates resulted in 4 as being the most likely number of genetically distinct groups in our data, estimated from ΔK (Figure 2). However, the 9-loci concatenate also showed a high support for K = 8, although a bit lower than K = 4 (Figure S2). In these cases, Evanno *et al*. [49] recommend using the smallest value of K because it represents the major structure in the data. Figure 1 also shows the 4 clusters detected by Structure mapped onto the phylogenetic tree.

To further characterize the genetic divergence of the four groups detected by Structure, we computed population pairwise F_ST_’s from haplotype frequencies with Arlequin (Table 2). Using the nomenclature defined in Table 2, the test resulted in group 4 (containing two of the most abundant STs in our data, ST-777 and ST-1358, that differ only in the *neuA* allele) being the most different with respect to the other three. Also, group 2 was more than 30% genetically different from groups 1 and 3, leaving these two groups being the most similar, as can also be seen in the phylogenetic reconstruction.

**Table 2:** Pairwise comparison between populations defined by Structure calculated with Arlequin.

	G1	G2	G3	G4
**G1**	0.933	0.346***	0.153*	0.529***
**G2**	0.286	0.495	0.473***	0.629***
**G3**	0.200	0.419	0.667	0.827***
**G4**	0.486	0.705	0.619	0.095

Average numbers of pairwise differences within populations are shown in diagonal. Upper matrix represents population pairwise F_ST_ and lower matrix shows the corrected average pairwise differences.

* p-value<0.05;

** p-value<0.01;

*** p-value<0.001.

A hierarchical analysis of molecular variance (AMOVA) was performed using Arlequin for each of the 10 loci independently considering two populations, the one from BV and the other one from the rest of CV. Results (Table S5 in File S1) showed significant differentiation between the two groups considered (p-value<0.05) despite most F_ST_ values were below 0.10. The only exception corresponded to locus *pilE* in which 13.73% of the total genetic variability was found among the two populations.

### Neutrality Tests

A summary of the neutrality tests for each locus is shown in Table S6 in File S1. Tajima’s D values suggest an excess of polymorphisms at low frequencies in *mip*, *mompS* and L6, which is confirmed by D* and F* for the two coding regions, directing these excess of single polymorphisms to the external branches of the phylogeny. Fu’s Fs gives evidence for an excess in the number of alleles in *mip*, as would be expected from a recent population expansion or genetic hitch-hiking. However, after FDR correction, only the intergenic L14 region was detected as significantly departed from neutralism by two of the tests (D and F*), and Fu’s Fs also rejected the null hypothesis of neutral evolution in the *neuA* genic fragment. Fu & Li’s D* was not able to reject neutralism in any of the 10 loci. So, although signs of selection or demographic effects were found especially in *mip*, no significant evidence of deviation from neutralism was finally found, neither truly demographic effects, in which case all loci would be affected similarly.

## Discussion

We have previously studied in detail the genetic variability and population structure of clinical and environmental isolates of *Legionella pneumophila* from different points of Comunidad Valenciana [15], [17], [53]. In this work, our main objective has been to analyze the extent and distribution of genetic variability in the environment of this species at a smaller geographic scale. For this, we have analyzed samples in a small area within the Comunidad Valenciana in which the water distribution systems of the sampled localities are supplied from the same watershed.

Although it has been known for a long time that the main natural habitats of *L. pneumophila* are freshwater environments such as rivers, lakes, ponds, and springs [54], [55], there have been very few studies addressed at characterizing the genetic variation of this species in these systems. Instead, most similar efforts have been devoted to characterize the diverse *Legionella* spp. present in different environments [56]–[59]. One recent study by Parthuisot *et al*. [60] analyzed the spatial and temporal dynamics of *Legionella* spp. in a French river watershed subject to seasonal and anthropogenic changes along the year. These authors found a higher prevalence of *L. pneumophila* over other species from the same genus, a result consistent with similar observations from other natural water environments [57], [61]. *Legionella* spp. colonize urban distribution systems were they can survive despite disinfection and control measures undertaken. Several studies have revealed a high prevalence of *L. pneumophila* also in these artificial environments [62] both before and after treatment. Despite our efforts, we have not been able to find any publication reporting on the genetic variation of *L. pneumophila* in different locations of the same watershed. Nevertheless, some studies in other water-borne human pathogens such as *E. coli* [63]–[65] and *Listeria monocytogenes* [66] have been performed with a similar approach.

Genetic variability in the *L. pneumophila* loci analyzed here was higher in the whole Comunidad Valenciana region than in the reduced BV area. These results are expected, because the CV dataset includes more distant localities and with different water-supply sources than the BV group. However, these general results do not apply to all the loci considered in the analysis, due to the unusual diversity pattern in locus *neuA* which results from the presence in some samples from the BV population of a particular group of alleles that corresponds to an alternative *neuA* gene from the one found in most serogroup 1 isolates [20].

The study of recombination in the haplotypes derived from the concatenate alignment of the 10-loci considered revealed four main events that were detected as statistically significant by six or even seven of the detection methods used. These events involved mainly loci *proA*, *pilE*, *neuA, mip, asd* and L6, either as separate loci or as a combination of at least two of them, as in *proA*+*pilE*, *neuA*+*mip* and *asd*+L6. These results are congruent with those obtained by Coscollá *et al*. [15] on a similar population from the Comunidad Valenciana, and also with the role that homologous recombination and horizontal gene transfer have had on the evolution of *L. pneumophila* [67], [68].

Recombination must be taken into account to understand the population structure of bacterial species, as recently shown in populations of *Salmonella enterica* [69]. In the present work, we have used isolates from 24 different geographical locations, but the phylogenetic reconstruction and analysis of the 30 resulting STs showed that their genetic composition was largely independent of their sampling location, except for group 4 (which only includes samples with the new *neuA* alleles) which was found only in the BV dataset. The four genetically distinct groups detected using Structure could be easily mapped onto the phylogenetic tree of all the loci, both when locus *neuA* was included (Figure 1) or not (Figure S1) in the concatenated alignment. Average population pairwise differences between the four genetic groups resulted in levels of diversity within groups comparable to those of some pairwise comparisons (Table 2).

Given that groups 1, 2 and 3 include isolates from both datasets, this result points to lack of genetic differentiation between BV and CV populations. However, we have found clear evidence of genetic differentiation between these two groups when the 10 loci were compared independently (Table S5 in File S1). In this case, the highest percentage of variability was found within populations, which held more than 90% of the total genetic variation in all loci except for *pilE*. Additionally, except for the ubiquitous ST-1 (strain Paris) [70], only 4 of the remaining 29 STs found were present in at least one population of each datasets. Therefore, there is some evidence of genetic differentiation between the BV and CV datasets which is somewhat disguised by the highly frequent presence of ST-1 in both groups.

The results derived from the present study give further insight into the population genetics of *L. pneumophila*, both at the macro and micro-environmental level. Despite the ubiquity of the most common genetic variant, ST-1, in the two datasets considered, we have found some evidence of genetic differentiation between them which cannot be attributed only to the presence in BV of some divergent alleles in locus *neuA*. We have confirmed that recombination likely plays an important role in shaping the genetic variability of this bacterium, and also in the independent evolution of different genes within the whole genome [67], [68]. However, further studies with complete genomes and more detailed samplings at different geographic scales are needed to draw more conclusions about the effect of selection and demographic events on the distribution of *L. pneumophila* in the environment.

## Supporting Information

Figure S1 ML phylogenetic reconstruction of the 9-loci alignment from data of the 133 environmental isolates from Vega Baixa (L) and the rest of Comunidad Valenciana (E) using RAxML. Bootstrap support values higher than 80% are shown.
(PDF)

Figure S2 Delta K values calculated by Evanno’s method using the 9-loci data by Structure Harvester Online.
(PDF)

File S1 Table S1, List of sequence types assigned to the 133 samples included in the study. The first letter in each sample name denotes the population group of origin: BV (L), rest of Comunidad Valenciana (E). Table S2, Distribution of *L. pneumophila* sequence types (STs) found in localities of the Comunidad Valenciana (Spain) and the BV area. Table S3, Summary of topological congruence tests for each locus tree and alignment. Summary of the p-values given by Shimodaira-Hasegawa (SH), Expected Likelihood Weight (ELW) and Approximately Unbiased (AU) tests using TREE-PUZZLE and CONSEL. Non-shadowed cells represent topological incongruence by rejection of the null hypothesis of the likelihood of the topology and the corresponding alignment being significantly different (p-value <0.05). cat9 and cat10 account for the 9-loci and 10-loci concatenates respectively. Table S4,Recombination events detected by RDP3. Colors represent the number of methods that significantly detect each of the events. Haplotypes in blank/grey distinguish between different clades on the phylogenetic tree. Table S5, Analysis of Molecular Variance for each locus. AMOVAs were performed with Arlequin. Levels of diversity explained by the variation among and within populations comparing the BV and CV datasets are shown. (d.f.: degrees of freedom). Table S6. Summary of neutrality tests. Tests performed with DnaSP for the 10 loci of all samples included in the study. Shadowed cells indicate significant deviation from neutrality after multiple-testing correction using FDR (α = 0.025).
(DOC)
