# Supplementary figures and images for: Genetic Characterization of Legionella pneumophila Isolated from a Common Watershed in Comunidad Valenciana, Spain
Source: PLoS One. 2013 Apr 25;8(4):e61564. doi: 10.1371/journal.pone.0061564 (PMC3636276; doi:10.1371/journal.pone.0061564)

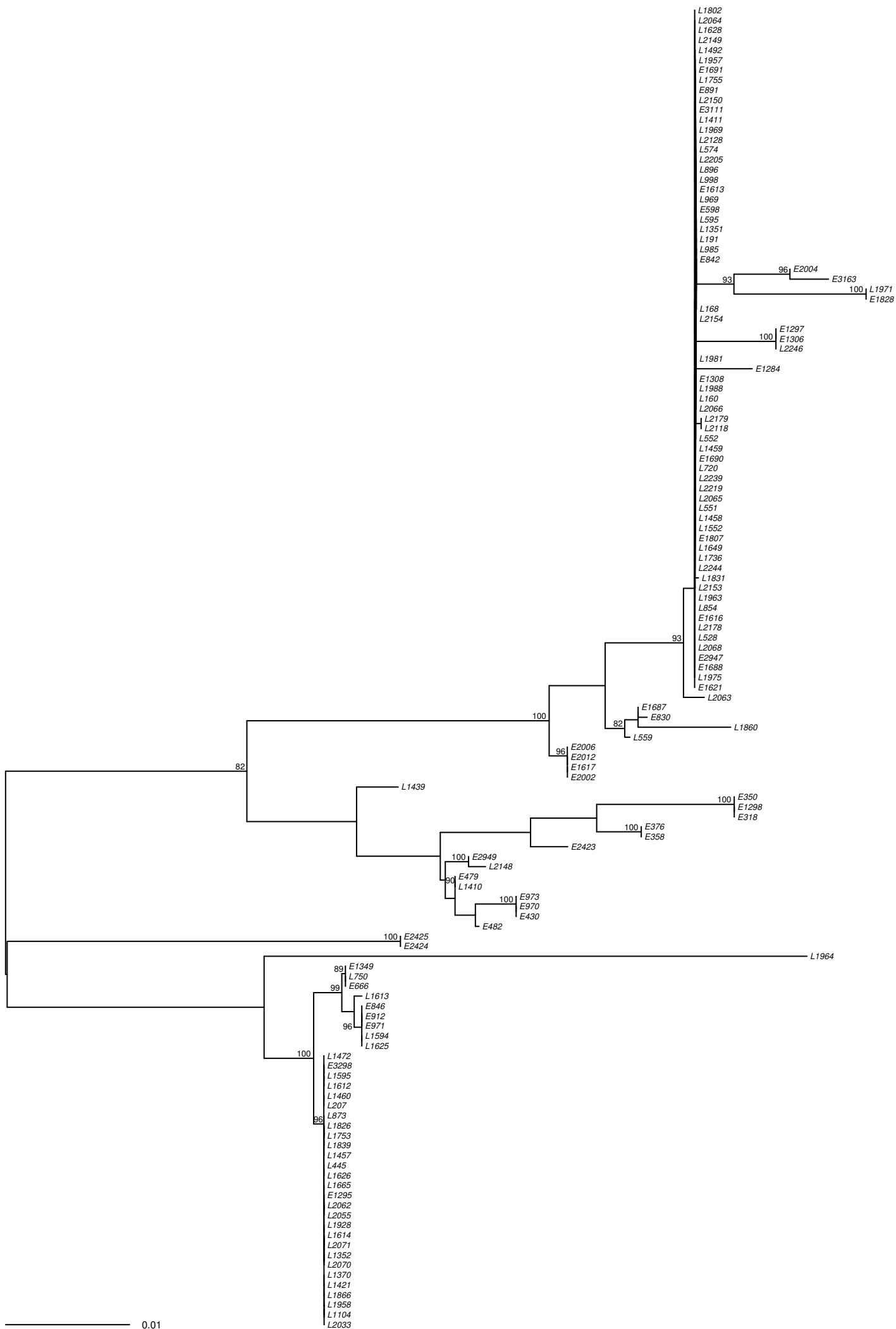

Supplement: Figure S1 — ML phylogenetic reconstruction of the 9-loci alignment from data of the 133 environmental isolates from Vega Baixa (L) and the rest of Comunidad Valenciana (E) using RAxML. Bootstrap support values higher than 80% are shown. (PDF) [file pone.0061564.s001.pdf]

$$\text{DeltaK} = \text{mean}(|L''(K)|) / \text{sd}(L(K))$$

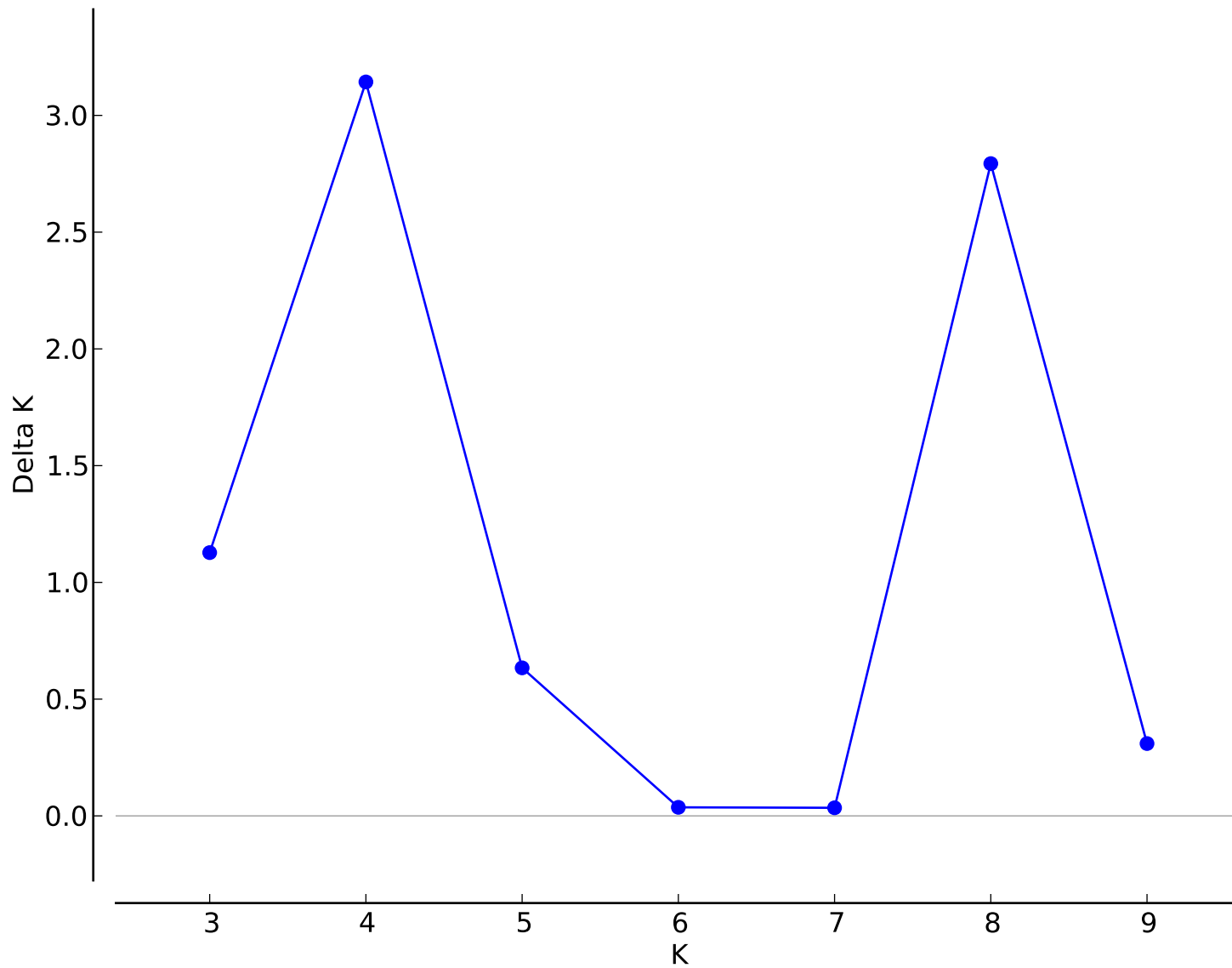

Supplement: Figure S2 — Delta K values calculated by Evanno’s method using the 9-loci data by Structure Harvester Online. (PDF) [file pone.0061564.s002.pdf]
