# Supplementary material for: Genetic Characterization of Legionella pneumophila Isolated from a Common Watershed in Comunidad Valenciana, Spain
Source: PLoS One. 2013 Apr 25;8(4):e61564. doi: 10.1371/journal.pone.0061564 (PMC3636276; doi:10.1371/journal.pone.0061564)
Supplement: File S1 — Table S1, List of sequence types assigned to the 133 samples included in the study. The first letter in each sample name denotes the population group of origin: BV (L), rest of Comunidad Valenciana (E). Table S2, Distribution of L. pneumophila sequence types (STs) found in localities of the Comunidad Valenciana (Spain) and the BV area. Table S3, Summary of topological congruence tests for each locus tree and alignment. Summary of the p-values given by Shimodaira-Hasegawa (SH), Expected Likelihood Weight (ELW) and Approximately Unbiased (AU) tests using TREE-PUZZLE and CONSEL. Non-shadowed cells represent topological incongruence by rejection of the null hypothesis of the likelihood of the topology and the corresponding alignment being significantly different (p-value <0.05). cat9 and cat10 account for the 9-loci and 10-loci concatenates respectively. Table S4,Recombination events detected by RDP3. Colors represent the number of methods that significantly detect each of the events. Haplotypes in blank/grey distinguish between different clades on the phylogenetic tree. Table S5, Analysis of Molecular Variance for each locus. AMOVAs were performed with Arlequin. Levels of diversity explained by the variation among and within populations comparing the BV and CV datasets are shown. (d.f.: degrees of freedom). Table S6. Summary of neutrality tests. Tests performed with DnaSP for the 10 loci of all samples included in the study. Shadowed cells indicate significant deviation from neutrality after multiple-testing correction using FDR (α = 0.025). (DOC) [file pone.0061564.s003.doc]

**Table S1.** List of sequence types assigned to the 133 samples included in the study. The first letter in each sample name denotes the population group of origin: BV (L), rest of Comunidad Valenciana (E).

| **Sample** | ***fliC*** | ***pilE*** | ***asd*** | ***mip*** | ***mompS*** | ***proA*** | ***neuA*** | **ST** |
| --- | --- | --- | --- | --- | --- | --- | --- | --- |
| **L1351** | 1 | 4 | 3 | 1 | 1 | 1 | 1 | **1** |
| **L1411** | 1 | 4 | 3 | 1 | 1 | 1 | 1 | **1** |
| **L1458** | 1 | 4 | 3 | 1 | 1 | 1 | 1 | **1** |
| **L1459** | 1 | 4 | 3 | 1 | 1 | 1 | 1 | **1** |
| **L1492** | 1 | 4 | 3 | 1 | 1 | 1 | 1 | **1** |
| **L1552** | 1 | 4 | 3 | 1 | 1 | 1 | 1 | **1** |
| **L160** | 1 | 4 | 3 | 1 | 1 | 1 | 1 | **1** |
| **L1628** | 1 | 4 | 3 | 1 | 1 | 1 | 1 | **1** |
| **L1649** | 1 | 4 | 3 | 1 | 1 | 1 | 1 | **1** |
| **L168** | 1 | 4 | 3 | 1 | 1 | 1 | 1 | **1** |
| **L1736** | 1 | 4 | 3 | 1 | 1 | 1 | 1 | **1** |
| **L1755** | 1 | 4 | 3 | 1 | 1 | 1 | 1 | **1** |
| **L1802** | 1 | 4 | 3 | 1 | 1 | 1 | 1 | **1** |
| **L1831** | 1 | 4 | 3 | 1 | 1 | 1 | 1 | **1** |
| **L191** | 1 | 4 | 3 | 1 | 1 | 1 | 1 | **1** |
| **L1957** | 1 | 4 | 3 | 1 | 1 | 1 | 1 | **1** |
| **L1963** | 1 | 4 | 3 | 1 | 1 | 1 | 1 | **1** |
| **L1969** | 1 | 4 | 3 | 1 | 1 | 1 | 1 | **1** |
| **L1971** | 1 | 4 | 3 | 1 | 1 | 1 | 1 | **1** |
| **L1975** | 1 | 4 | 3 | 1 | 1 | 1 | 1 | **1** |
| **L1981** | 1 | 4 | 3 | 1 | 1 | 1 | 1 | **1** |
| **L1988** | 1 | 4 | 3 | 1 | 1 | 1 | 1 | **1** |
| **L2064** | 1 | 4 | 3 | 1 | 1 | 1 | 1 | **1** |
| **L2065** | 1 | 4 | 3 | 1 | 1 | 1 | 1 | **1** |
| **L2066** | 1 | 4 | 3 | 1 | 1 | 1 | 1 | **1** |
| **L2068** | 1 | 4 | 3 | 1 | 1 | 1 | 1 | **1** |
| **L2118** | 1 | 4 | 3 | 1 | 1 | 1 | 1 | **1** |
| **L2128** | 1 | 4 | 3 | 1 | 1 | 1 | 1 | **1** |
| **L2149** | 1 | 4 | 3 | 1 | 1 | 1 | 1 | **1** |
| **L2150** | 1 | 4 | 3 | 1 | 1 | 1 | 1 | **1** |
| **L2153** | 1 | 4 | 3 | 1 | 1 | 1 | 1 | **1** |
| **L2154** | 1 | 4 | 3 | 1 | 1 | 1 | 1 | **1** |
| **L2178** | 1 | 4 | 3 | 1 | 1 | 1 | 1 | **1** |
| **L2179** | 1 | 4 | 3 | 1 | 1 | 1 | 1 | **1** |
| **L2205** | 1 | 4 | 3 | 1 | 1 | 1 | 1 | **1** |
| **L2219** | 1 | 4 | 3 | 1 | 1 | 1 | 1 | **1** |
| **L2239** | 1 | 4 | 3 | 1 | 1 | 1 | 1 | **1** |
| **L2244** | 1 | 4 | 3 | 1 | 1 | 1 | 1 | **1** |
| **L528** | 1 | 4 | 3 | 1 | 1 | 1 | 1 | **1** |
| **L552** | 1 | 4 | 3 | 1 | 1 | 1 | 1 | **1** |
| **L574** | 1 | 4 | 3 | 1 | 1 | 1 | 1 | **1** |
| **L595** | 1 | 4 | 3 | 1 | 1 | 1 | 1 | **1** |
| **L720** | 1 | 4 | 3 | 1 | 1 | 1 | 1 | **1** |
| **L854** | 1 | 4 | 3 | 1 | 1 | 1 | 1 | **1** |
| **L896** | 1 | 4 | 3 | 1 | 1 | 1 | 1 | **1** |
| **L969** | 1 | 4 | 3 | 1 | 1 | 1 | 1 | **1** |
| **L998** | 1 | 4 | 3 | 1 | 1 | 1 | 1 | **1** |
| **L551** | 1 | 4 | 3 | 1 | 1 | 1 | 9 | **8** |
| **L985** | 1 | 4 | 3 | 1 | 1 | 1 | 9 | **8** |
| **L559** | 2 | 3 | 6 | 10 | 2 | 1 | 6 | **22** |
| **L1613** | 5 | 1 | 22 | 26 | 6 | 10 | 12 | **45** |
| **L1594** | 5 | 2 | 22 | 27 | 6 | 10 | 12 | **48** |
| **L1625** | 5 | 2 | 22 | 27 | 6 | 10 | 12 | **48** |
| **L2246** | 1 | 4 | 3 | 19 | 1 | 1 | 1 | **719** |
| **L2062** | 5 | 2 | 22 | 10 | 6 | 25 | 1 | **777** |
| **L207** | 5 | 2 | 22 | 10 | 6 | 25 | 1 | **777** |
| **L2071** | 5 | 2 | 22 | 10 | 6 | 25 | 1 | **777** |
| **L445** | 5 | 2 | 22 | 10 | 6 | 25 | 1 | **777** |
| **L873** | 5 | 2 | 22 | 10 | 6 | 25 | 1 | **777** |
| **L750** | 5 | 1 | 22 | 30 | 6 | 10 | 1 | **856** |
| **L2063** | 1 | 4 | 3 | 1 | 6 | 1 | 1 | **857** |
| **L2148** | 2 | 10 | 19 | 44 | 19 | 4 | 36 | **858** |
| **L1964** | 6 | 41 | 45 | 51 | 55 | 10 | 31 | **864** |
| **L1410** | 6 | 10 | 15 | 3 | 21 | 4 | 207 | **1356** |
| **L1439** | 2 | 10 | 3 | 16 | 9 | 4 | 208 | **1357** |
| **L1860** | 4 | 3 | 18 | 10 | 5 | 1 | 218 | **1374** |
| **L1104** | 5 | 2 | 22 | 10 | 6 | 25 | 203 | **1358** |
| **L1352** | 5 | 2 | 22 | 10 | 6 | 25 | 203 | **1358** |
| **L1370** | 5 | 2 | 22 | 10 | 6 | 25 | 203 | **1358** |
| **L1421** | 5 | 2 | 22 | 10 | 6 | 25 | 203 | **1358** |
| **L1457** | 5 | 2 | 22 | 10 | 6 | 25 | 203 | **1358** |
| **L1460** | 5 | 2 | 22 | 10 | 6 | 25 | 203 | **1358** |
| **L1472** | 5 | 2 | 22 | 10 | 6 | 25 | 203 | **1358** |
| **L1595** | 5 | 2 | 22 | 10 | 6 | 25 | 203 | **1358** |
| **L1612** | 5 | 2 | 22 | 10 | 6 | 25 | 203 | **1358** |
| **L1614** | 5 | 2 | 22 | 10 | 6 | 25 | 203 | **1358** |
| **L1626** | 5 | 2 | 22 | 10 | 6 | 25 | 203 | **1358** |
| **L1665** | 5 | 2 | 22 | 10 | 6 | 25 | 203 | **1358** |
| **L1753** | 5 | 2 | 22 | 10 | 6 | 25 | 203 | **1358** |
| **L1826** | 5 | 2 | 22 | 10 | 6 | 25 | 203 | **1358** |
| **L1839** | 5 | 2 | 22 | 10 | 6 | 25 | 203 | **1358** |
| **L1866** | 5 | 2 | 22 | 10 | 6 | 25 | 203 | **1358** |
| **L1928** | 5 | 2 | 22 | 10 | 6 | 25 | 203 | **1358** |
| **L1958** | 5 | 2 | 22 | 10 | 6 | 25 | 203 | **1358** |
| **L2033** | 5 | 2 | 22 | 10 | 6 | 25 | 203 | **1358** |
| **L2055** | 5 | 2 | 22 | 10 | 6 | 25 | 203 | **1358** |
| **L2070** | 5 | 2 | 22 | 10 | 6 | 25 | 203 | **1358** |
| **E1284** | 1 | 4 | 3 | 1 | 1 | 1 | 1 | **1** |
| **E1308** | 1 | 4 | 3 | 1 | 1 | 1 | 1 | **1** |
| **E1613** | 1 | 4 | 3 | 1 | 1 | 1 | 1 | **1** |
| **E1616** | 1 | 4 | 3 | 1 | 1 | 1 | 1 | **1** |
| **E1621** | 1 | 4 | 3 | 1 | 1 | 1 | 1 | **1** |
| **E1688** | 1 | 4 | 3 | 1 | 1 | 1 | 1 | **1** |
| **E1690** | 1 | 4 | 3 | 1 | 1 | 1 | 1 | **1** |
| **E1691** | 1 | 4 | 3 | 1 | 1 | 1 | 1 | **1** |
| **E1807** | 1 | 4 | 3 | 1 | 1 | 1 | 1 | **1** |
| **E1828** | 1 | 4 | 3 | 1 | 1 | 1 | 1 | **1** |
| **E2004** | 1 | 4 | 3 | 1 | 1 | 1 | 1 | **1** |
| **E2947** | 1 | 4 | 3 | 1 | 1 | 1 | 1 | **1** |
| **E3111** | 1 | 4 | 3 | 1 | 1 | 1 | 1 | **1** |
| **E598** | 1 | 4 | 3 | 1 | 1 | 1 | 1 | **1** |
| **E842** | 1 | 4 | 3 | 1 | 1 | 1 | 1 | **1** |
| **E891** | 1 | 4 | 3 | 1 | 1 | 1 | 1 | **1** |
| **E830** | 2 | 3 | 18 | 15 | 2 | 1 | 6 | **20** |
| **E846** | 5 | 2 | 22 | 27 | 6 | 10 | 12 | **48** |
| **E912** | 5 | 2 | 22 | 27 | 6 | 10 | 12 | **48** |
| **E971** | 5 | 2 | 22 | 27 | 6 | 10 | 12 | **48** |
| **E666** | 5 | 1 | 22 | 30 | 6 | 10 | 6 | **74** |
| **E1617** | 2 | 10 | 18 | 10 | 2 | 1 | 6 | **146** |
| **E2002** | 2 | 10 | 18 | 10 | 2 | 1 | 6 | **146** |
| **E2006** | 2 | 10 | 18 | 10 | 2 | 1 | 6 | **146** |
| **E2012** | 2 | 10 | 18 | 10 | 2 | 1 | 6 | **146** |
| **E479** | 6 | 10 | 15 | 3 | 21 | 4 | 9 | **171** |
| **E2424** | 3 | 10 | 1 | 12 | 14 | 9 | 9 | **181** |
| **E2425** | 3 | 10 | 1 | 12 | 14 | 9 | 9 | **181** |
| **E2949** | 6 | 10 | 19 | 28 | 19 | 4 | 9 | **328** |
| **E1298** | 6 | 10 | 17 | 3 | 4 | 14 | 9 | **374** |
| **E318** | 6 | 10 | 17 | 3 | 4 | 14 | 9 | **374** |
| **E350** | 6 | 10 | 17 | 3 | 4 | 14 | 9 | **374** |
| **E358** | 2 | 10 | 3 | 28 | 21 | 4 | 13 | **464** |
| **E376** | 2 | 10 | 3 | 28 | 21 | 4 | 13 | **464** |
| **E1297** | 1 | 4 | 3 | 19 | 1 | 1 | 1 | **719** |
| **E3298** | 5 | 2 | 22 | 10 | 6 | 25 | 9 | **745** |
| **E1295** | 5 | 2 | 22 | 10 | 6 | 25 | 1 | **777** |
| **E482** | 6 | 10 | 15 | 3 | 21 | 14 | 6 | **804** |
| **E1349** | 5 | 1 | 22 | 30 | 6 | 10 | 1 | **856** |
| **E1687** | 2 | 3 | 18 | 10 | 2 | 1 | 1 | **1035** |
| **E1306** | 1 | 4 | 3 | 19 | 1 | 1 | 3 | **1036** |
| **E2423** | 6 | 10 | 14 | 28 | 21 | 4 | 9 | **1037** |
| **E3163** | 1 | 6 | 3 | 10 | 1 | 5 | 11 | **1038** |
| **E430** | 6 | 10 | 15 | 13 | 21 | 14 | 6 | **1039** |
| **E970** | 6 | 10 | 15 | 13 | 21 | 14 | 6 | **1039** |
| **E973** | 6 | 10 | 15 | 13 | 21 | 14 | 6 | **1039** |

**Table S2.** Distribution of *L. pneumophila* sequence types (STs) found in localities of the Comunidad Valenciana (Spain) and the BV area.

|  | Sequence Type | | | | | | | | | | | | | | | | | | | | | | | | | | | | | |
| --- | --- | --- | --- | --- | --- | --- | --- | --- | --- | --- | --- | --- | --- | --- | --- | --- | --- | --- | --- | --- | --- | --- | --- | --- | --- | --- | --- | --- | --- | --- |
| Location | 1 | 8 | 20 | 22 | 45 | 48 | 74 | 146 | 171 | 181 | 328 | 374 | 464 | 719 | 745 | 777 | 804 | 856 | 857 | 858 | 864 | 1035 | 1036 | 1037 | 1038 | 1039 | 1356 | 1357 | 1374 | 1358 |
| CV-1 | 6 |  |  |  |  | 2 |  |  | 1 | 2 | 1 |  |  |  | 1 |  | 1 |  |  |  |  | 1 | 1 | 1 | 1 | 1 |  |  |  |  |
| CV-2 | 1 |  |  |  |  |  |  |  |  |  |  |  |  |  |  |  |  |  |  |  |  |  |  |  |  |  |  |  |  |  |
| CV-3 | 2 |  | 1 |  |  | 1 | 1 |  |  |  |  | 2 | 2 |  |  |  |  |  |  |  |  |  |  |  |  | 1 |  |  |  |  |
| CV-4 | 1 |  |  |  |  |  |  |  |  |  |  |  |  |  |  |  |  |  |  |  |  |  |  |  |  |  |  |  |  |  |
| CV-5 |  |  |  |  |  |  |  |  |  |  |  | 1 |  | 1 |  | 1 |  | 1 |  |  |  |  |  |  |  |  |  |  |  |  |
| CV-6 | 2 |  |  |  |  |  |  |  |  |  |  |  |  |  |  |  |  |  |  |  |  |  |  |  |  | 1 |  |  |  |  |
| CV-7 | 4 |  |  |  |  |  |  | 4 |  |  |  |  |  |  |  |  |  |  |  |  |  |  |  |  |  |  |  |  |  |  |
| BV-1 | 11 |  |  |  | 1 |  |  |  |  |  |  |  |  | 1 |  | 3 |  | 1 | 1 | 1 |  |  |  |  |  |  | 9 |  |  |  |
| BV-2 | 7 |  |  |  |  | 2 |  |  |  |  |  |  |  |  |  |  |  |  |  |  |  |  |  |  |  |  | 6 |  | 1 | 1 |
| BV-3 | 4 | 1 |  |  |  |  |  |  |  |  |  |  |  |  |  |  |  |  |  |  |  |  |  |  |  |  | 2 | 1 |  |  |
| BV-4 | 3 |  |  |  |  |  |  |  |  |  |  |  |  |  |  |  |  |  |  |  |  |  |  |  |  |  | 2 |  |  |  |
| BV-5 | 3 |  |  |  |  |  |  |  |  |  |  |  |  |  |  | 1 |  |  |  |  |  |  |  |  |  |  |  |  |  |  |
| BV-6 | 6 |  |  | 1 |  |  |  |  |  |  |  |  |  |  |  | 1 |  |  |  |  |  |  |  |  |  |  | 1 |  |  |  |
| BV-7 | 2 |  |  |  |  |  |  |  |  |  |  |  |  |  |  |  |  |  |  |  |  |  |  |  |  |  |  |  |  |  |
| BV-8 | 3 |  |  |  |  |  |  |  |  |  |  |  |  |  |  |  |  |  |  |  |  |  |  |  |  |  |  |  |  |  |
| BV-9 | 4 |  |  |  |  |  |  |  |  |  |  |  |  |  |  |  |  |  |  |  |  |  |  |  |  |  | 1 |  |  |  |
| BV-10 | 1 |  |  |  |  |  |  |  |  |  |  |  |  |  |  |  |  |  |  |  |  |  |  |  |  |  |  |  |  |  |
| BV-11 | 2 |  |  |  |  |  |  |  |  |  |  |  |  |  |  |  |  |  |  |  | 1 |  |  |  |  |  |  |  |  |  |
| BV-13 | 1 |  |  |  |  |  |  |  |  |  |  |  |  |  |  |  |  |  |  |  |  |  |  |  |  |  |  |  |  |  |
| BV-14 |  | 1 |  |  |  |  |  |  |  |  |  |  |  |  |  |  |  |  |  |  |  |  |  |  |  |  |  |  |  |  |
| Total | 63 | 2 | 1 | 1 | 1 | 5 | 1 | 4 | 1 | 2 | 1 | 3 | 2 | 2 | 1 | 6 | 1 | 2 | 1 | 1 | 1 | 1 | 1 | 1 | 1 | 3 | 21 | 1 | 1 | 1 |

**Table S3.** Summary of the p-values given by Shimodaira-Hasegawa (SH), Expected Likelihood Weight (ELW) and Approximately Unbiased (AU) tests using TREE-PUZZLE and CONSEL. Non-shadowed cells represent topological incongruence by rejection of the null hypothesis of the likelihood of the topology and the corresponding alignment being significantly different (p-value < 0.05). cat9 and cat10 account for the 9-loci and 10-loci concatenates respectively.

| **Tree** | **L14** | | | ***proA*** | | | ***pilE*** | | | **L2** | | | ***neuA*** | | | ***mip*** | | |
| --- | --- | --- | --- | --- | --- | --- | --- | --- | --- | --- | --- | --- | --- | --- | --- | --- | --- | --- |
|  | **SH** | **ELW** | **AU** | **SH** | **ELW** | **AU** | **SH** | **ELW** | **AU** | **SH** | **ELW** | **AU** | **SH** | **ELW** | **AU** | **SH** | **ELW** | **AU** |
| L14 | 1.0000 | 1.0000 | 1.0000 | 0.2760 | 0.0000 | 2.00E-05 | 0.1580 | 0.0000 | 5.00E-109 | 0.0080 | 0.0000 | 9.00E-48 | 0.0000 | 0.0000 | 9.00E-05 | 0.0000 | 0.0000 | 6.00E-63 |
| *proA* | 0.1980 | 0.0000 | 3.00E-65 | 1.0000 | 0.9897 | 0.9960 | 0.0000 | 0.0000 | 5.00E-07 | 0.0000 | 0.0000 | 4.00E-05 | 0.0000 | 0.0000 | 4.00E-26 | 0.0030 | 0.0000 | 8.00E-60 |
| *pilE* | 0.0020 | 0.0000 | 4.00E-48 | 0.0010 | 0.0000 | 2.00E-04 | 1.0000 | 1.0000 | 0.9990 | 0.0000 | 0.0000 | 5.00E-08 | 0.0000 | 0.0000 | 1.00E-05 | 0.0000 | 0.0000 | 4.00E-70 |
| L2 | 0.0030 | 0.0000 | 3.00E-06 | 0.0900 | 0.0000 | 9.00E-05 | 0.0020 | 0.0000 | 8.00E-05 | 1.0000 | 1.0000 | 1.0000 | 0.0000 | 0.0000 | 1.00E-05 | 0.0000 | 0.0000 | 6.00E-59 |
| *neuA* | 0.0000 | 0.0000 | 2.00E-04 | 0.0020 | 0.0000 | 6.00E-16 | 0.0000 | 0.0000 | 5.00E-40 | 0.0000 | 0.0000 | 2.00E-10 | 1.0000 | 1.0000 | 1.0000 | 0.0000 | 0.0000 | 9.00E-89 |
| *mip* | 0.0040 | 0.0000 | 2.00E-05 | 0.2060 | 0.0000 | 4.00E-67 | 0.0230 | 0.0000 | 1.00E-81 | 0.0000 | 0.0000 | 6.00E-67 | 0.0000 | 0.0000 | 8.00E-05 | 1.0000 | 1.0000 | 1.0000 |
| *fliC* | 0.0030 | 0.0000 | 1.00E-39 | 0.0930 | 0.0000 | 1.00E-19 | 0.0440 | 0.0000 | 2.00E-51 | 0.0000 | 0.0000 | 4.00E-82 | 0.0000 | 0.0000 | 9.00E-07 | 0.0000 | 0.0000 | 2.00E-54 |
| L6 | 0.0270 | 0.0000 | 1.00E-53 | 0.1660 | 0.0000 | 4.00E-09 | 0.0130 | 0.0000 | 2.00E-99 | 0.0000 | 0.0000 | 2.00E-219 | 0.0000 | 0.0000 | 4.00E-05 | 0.0000 | 0.0000 | 5.00E-82 |
| *asd* | 0.0060 | 0.0000 | 6.00E-52 | 0.2940 | 0.0000 | 3.00E-04 | 0.0000 | 0.0000 | 3.00E-50 | 0.0030 | 0.0000 | 4.00E-05 | 0.0000 | 0.0000 | 1.00E-64 | 0.0000 | 0.0000 | 9.00E-49 |
| *mompS* | 0.0230 | 0.0000 | 0.0010 | 0.1650 | 0.0000 | 8.00E-05 | 0.0480 | 0.0000 | 2.00E-06 | 0.0000 | 0.0000 | 1.00E-10 | 0.0000 | 0.0000 | 4.00E-61 | 0.0000 | 0.0000 | 2.00E-69 |
| cat10 | 0.1630 | 0.0000 | 3.00E-37 | 0.3170 | 0.0000 | 2.00E-04 | 0.2070 | 0.0000 | 1.00E-04 | 0.0760 | 0.0000 | 5.00E-09 | 0.3020 | 0.0000 | 3.00E-72 | 0.1010 | 0.0000 | 4.00E-112 |
| cat9 | 0.4570 | 0.0000 | 1.00E-87 | 0.6960 | 0.0103 | 0.0040 | 0.4170 | 0.0000 | 0.0010 | 0.2610 | 0.0000 | 5.00E-35 | 0.0000 | 0.0000 | 1.00E-04 | 0.2700 | 0.0000 | 3.00E-52 |
|  |  |  |  |  |  |  |  |  |  |  |  |  |  |  |  |  |  |  |
|  |  |  |  |  |  |  |  |  |  |  |  |  |  |  |  |  |  |  |
| **Tree** | ***fliC*** | | | **L6** | | | ***asd*** | | | ***mompS*** | | | **cat10** | | | **cat9** | | |
|  | **SH** | **ELW** | **AU** | **SH** | **ELW** | **AU** | **SH** | **ELW** | **AU** | **SH** | **ELW** | **AU** | **SH** | **ELW** | **AU** | **SH** | **ELW** | **AU** |
| L14 | 0.5400 | 0.0045 | 0.0530 | 0.1330 | 0.0000 | 4.00E-06 | 0.2630 | 0.0000 | 4.00E-05 | 0.2460 | 0.0000 | 1.00E-05 | 0.0000 | 0.0000 | 1.00E-155 | 0.0860 | 0.0000 | 3.00E-04 |
| *proA* | 0.0110 | 0.0000 | 2.00E-06 | 0.0020 | 0.0000 | 1.00E-14 | 0.0190 | 0.0000 | 2.00E-07 | 0.0440 | 0.0000 | 9.00E-06 | 0.0000 | 0.0000 | 1.00E-95 | 0.0000 | 0.0000 | 2.00E-47 |
| *pilE* | 0.2970 | 0.0000 | 4.00E-53 | 0.0000 | 0.0000 | 0.0010 | 0.0260 | 0.0000 | 2.00E-09 | 0.0050 | 0.0000 | 1.00E-05 | 0.0000 | 0.0000 | 1.00E-59 | 0.0000 | 0.0000 | 2.00E-46 |
| L2 | 0.0460 | 0.0000 | 9.00E-05 | 0.0090 | 0.0000 | 8.00E-08 | 0.1410 | 0.0000 | 9.00E-05 | 0.0080 | 0.0000 | 3.00E-41 | 0.0000 | 0.0000 | 1.00E-69 | 0.0000 | 0.0000 | 5.00E-05 |
| *neuA* | 0.0010 | 0.0000 | 3.00E-05 | 0.0000 | 0.0000 | 2.00E-07 | 0.0030 | 0.0000 | 9.00E-09 | 0.0010 | 0.0000 | 2.00E-07 | 0.0000 | 0.0000 | 3.00E-45 | 0.0000 | 0.0000 | 2.00E-29 |
| *mip* | 0.2100 | 0.0000 | 9.00E-05 | 0.0000 | 0.0000 | 3.00E-09 | 0.0530 | 0.0000 | 2.00E-10 | 0.0480 | 0.0000 | 9.00E-81 | 0.0000 | 0.0000 | 2.00E-05 | 0.0000 | 0.0000 | 3.00E-06 |
| *fliC* | 1.0000 | 0.9832 | 1.0000 | 0.0000 | 0.0000 | 1.00E-05 | 0.1520 | 0.0000 | 1.00E-76 | 0.0530 | 0.0000 | 8.00E-36 | 0.0000 | 0.0000 | 5.00E-53 | 0.0000 | 0.0000 | 3.00E-09 |
| L6 | 0.2300 | 0.0000 | 4.00E-04 | 1.0000 | 1.0000 | 1.0000 | 0.1580 | 0.0000 | 8.00E-10 | 0.1850 | 0.0000 | 3.00E-05 | 0.0000 | 0.0000 | 2.00E-07 | 0.0020 | 0.0000 | 8.00E-77 |
| *asd* | 0.1460 | 0.0000 | 4.00E-14 | 0.0570 | 0.0000 | 1.00E-09 | 1.0000 | 1.0000 | 1.0000 | 0.0830 | 0.0000 | 1.00E-08 | 0.0000 | 0.0000 | 9.00E-10 | 0.0000 | 0.0000 | 4.00E-09 |
| *mompS* | 0.3960 | 0.0000 | 3.00E-42 | 0.1730 | 0.0000 | 2.00E-08 | 0.1300 | 0.0000 | 1.00E-11 | 1.0000 | 1.0000 | 1.0000 | 0.0000 | 0.0000 | 3.00E-05 | 0.0000 | 0.0000 | 1.00E-48 |
| cat10 | 0.4330 | 0.0000 | 1.00E-07 | 0.2730 | 0.0000 | 3.00E-44 | 0.2530 | 0.0000 | 2.00E-11 | 0.2970 | 0.0000 | 3.00E-56 | 1.0000 | 1.0000 | 1.0000 | 0.2120 | 0.0000 | 3.00E-50 |
| cat9 | 0.5780 | 0.0123 | 3.00E-04 | 0.1410 | 0.0000 | 1.00E-06 | 0.5670 | 0.0000 | 1.00E-06 | 0.2760 | 0.0000 | 6.00E-95 | 0.0000 | 0.0000 | 1.00E-79 | 1.0000 | 1.0000 | 1.0000 |

**Table S4.** Recombination events detected by RDP3. Colors represent the number of methods that significantly detect each of the events. Haplotypes in blank/grey distinguish between different clades on the phylogenetic tree.

| **Haplotypes** | **L14** | ***proA*** | ***pilE*** | **L2** | ***neuA*** | ***mip*** | ***fliC*** | **L6** | ***asd*** | ***mompS*** |  |
| --- | --- | --- | --- | --- | --- | --- | --- | --- | --- | --- | --- |
| cE430_cE970_cE973 |  |  | |  |  |  |  |  |  |  |  |
| cE479 |  |  | |  |  |  |  |  |  |  |  |
| cE482 |  |  | |  |  |  |  |  |  |  |  |
| cE2949 |  |  | |  |  |  |  |  |  |  |  |
| cE2423 |  |  | |  | | |  |  |  |  |  |
| cE1298_cE318_cE350 |  |  | |  | | |  |  |  |  |  |
| cE358_cE376 |  |  | |  | | |  |  | |  |  |
| cL2148 |  |  | |  |  | |  |  |  |  |  |
| cE2424_cE2425 |  |  |  | | |  |  |  |  |  |  |
| cE1308_cE1613_cE1616… |  |  |  |  |  | |  |  | |  |  |
| cE1284 |  |  |  |  |  | |  |  | |  |  |
| cL2063 |  |  |  |  |  | |  |  | |  |  |
| cL2118_cL2179 |  |  |  |  |  |  |  |  | |  |  |
| cE2004 |  |  |  |  |  |  |  |  | |  |  |
| cE1828_cL1971 |  |  |  |  |  |  |  |  | |  |  |
| cE1306 |  |  |  |  |  |  |  |  | |  |  |
| cE1297 |  |  |  |  |  |  |  |  | |  |  |
| cL2246 |  |  |  |  |  |  |  |  | |  |  |
| cL551_cL985 |  |  |  |  |  | |  |  | |  |  |
| cL1831 |  |  |  |  |  | |  |  | |  |  |
| cL559 |  |  |  |  |  | |  |  |  |  |  |
| cE1687 |  |  |  |  |  | |  |  |  |  |  |
| cE1617_cE2002_cE2006_cE2012 |  |  |  |  |  | |  |  |  |  |  |
| cE830 |  |  |  |  |  | |  |  |  |  |  |
|  |  |  |  |  | |  |  |  |  |  |
| cE3163 |  |  |  |  | |  |  |  | |  |  |
|  |  |  |  | | |  |  |  |  |  |
| cL1964 |  |  |  |  |  |  |  |  |  |  |  |
| cL1410 |  |  | |  |  |  |  |  |  |  |  |
| cL1439 |  |  | |  |  | |  |  | |  |  |
| cL1860 |  |  |  |  |  |  |  |  |  |  |  |
|  |  |  |  |  | |  |  |  |  |  |
| cL1104_cL1352_cL1370_cL1421… |  |  |  |  |  |  |  |  |  |  |  |
| cE1295_cL2062_cL207… |  |  |  |  |  |  |  |  |  |  |  |
| cE3298 |  |  |  |  |  | |  |  |  |  |  |
| cE1349_cL750 |  |  |  |  |  |  |  |  |  |  |  |
| cE666 |  |  |  |  |  |  |  |  |  |  |  |
| cL1613 |  |  |  |  |  |  |  |  |  |  |  |
| cE846_cE912_cE971_cL1594_cL1625 |  |  |  |  |  |  |  |  |  |  |  |

|  | 7 methods |
| --- | --- |
|  | 6 methods |
|  | 5 methods |
|  | 4 methods |
|  | 3 methods |
|  |  |

|  | **Source of variation** | **d.f.** | **Sum of squares** | **Variance components** | **Percentage of variation** | **Fixation Index (F_ST_)** | **Significance test (10000 perm.)** |
| --- | --- | --- | --- | --- | --- | --- | --- |
| **L14** | Among populations | 1 | 1.96600 | 0.02721 | 7.65000 | 0.07645 | 0.0099+-0.00030 |
|  | Within populations | 131 | 43.06400 | 0.32873 | 92.35000 |  |  |
|  | Total | 132 | 45.03000 | 0.35594 |  |  |  |
| ***proA*** | Among populations | 1 | 1.60300 | 0.02170 | 6.82000 | 0.06820 | 0.00168+-0.00042 |
|  | Within populations | 131 | 38.84100 | 0.29650 | 93.18000 |  |  |
|  | Total | 132 | 40.44400 |  |  |  |  |
| ***pilE*** | Among populations | 1 | 3.19200 | 0.04803 | 13.73000 | 0.13732 | 0.00000+-0.00000 |
|  | Within populations | 131 | 39.52900 | 0.30175 | 86.27000 |  |  |
|  | Total | 132 | 42.72200 | 0.34978 |  |  |  |
| **L2** | Among populations | 1 | 2.08200 | 0.02929 | 8.41000 | 0.08413 | 0.00129+-0.00033 |
|  | Within populations | 131 | 41.76800 | 0.31884 | 91.59000 |  |  |
|  | Total | 132 | 43.85000 | 0.34813 |  |  |  |
| ***neuA*** | Among populations | 1 | 2.38300 | 0.03440 | 9.92000 | 0.09921 | 0.00010+-0.00010 |
|  | Within populations | 131 | 40.91800 | 0.31235 | 90.08000 |  |  |
|  | Total | 132 | 43.30100 | 0.34675 |  |  |  |
| ***mip*** | Among populations | 1 | 2.39100 | 0.03414 | 9.19000 | 0.09193 | 0.00000+-0.00000 |
|  | Within populations | 131 | 44.17200 | 0.33719 | 90.81000 |  |  |
|  | Total | 132 | 46.56400 | 0.37133 |  |  |  |
| ***fliC*** | Among populations | 1 | 1.90100 | 0.02651 | 7.99000 | 0.07986 | 0.00218+-0.0046 |
|  | Within populations | 131 | 40.01600 | 0.30547 | 92.01000 |  |  |
|  | Total | 132 | 41.91700 | 0.33198 |  |  |  |
| **L6** | Among populations | 1 | 2.29200 | 0.03286 | 9.47000 | 0.09471 | 0.00010+-0.00010 |
|  | Within populations | 131 | 41.15200 | 0.31413 | 90.53000 |  |  |
|  | Total | 132 | 43.44400 | 0.34700 |  |  |  |
| ***asd*** | Among populations | 1 | 1.31800 | 0.01688 | 5.29000 | 0.05289 | 0.01050+-0.00085 |
|  | Within populations | 131 | 39.60600 | 0.30234 | 94.71000 |  |  |
|  | Total | 132 | 40.92500 | 0.31922 |  |  |  |
| ***mompS*** | Among populations | 1 | 1.54200 | 0.02028 | 5.93000 | 0.05931 | 0.00703+-0.00089 |
|  | Within populations | 131 | 42.13500 | 0.32164 | 94.07000 |  |  |
|  | Total | 132 | 43.67700 | 0.34192 |  |  |  |

**Table S5.** Analysis of Molecular Variance for each locus. AMOVAs were performed with Arlequin. Levels of diversity explained by the variation among and within populations comparing the BV and CV datasets are shown. (d.f.: degrees of freedom).

**Table S6.** Summary of neutrality tests performed with DnaSP for the 10 loci of all samples included in the study. Shadowed cells indicate significant deviation from neutrality after multiple-testing correction using FDR (α = 0.025).

| **Locus** | **Tajima's D** | | **Fu & Li's D*** | | **Fu & Li's F*** | | **Fu's Fs** | |
| --- | --- | --- | --- | --- | --- | --- | --- | --- |
| L14 | 2.821 | *** | 1.408 | * | 2.412 | *** | 20.748 | *** |
| *proA* | 1.658 | * | 1.717 | ** | 2.028 | * | 9.507 | * |
| *pilE* | 0.868 |  | 0.314 |  | 0.639 |  | 10.936 | ** |
| L2 | 1.103 |  | 0.784 |  | 1.103 |  | 16.468 | ** |
| *neuA* | 1.851 | * | 1.005 |  | 1.664 | * | 58.988 | *** |
| *mip* | -0.735 |  | -0.158 |  | -0.462 |  | -1.328 |  |
| *fliC* | 1.124 |  | 0.348 |  | 0.758 |  | 6.508 | * |
| L6 | -0.301 |  | 0.905 |  | 0.505 |  | 4.592 |  |
| *asd* | 1.362 |  | 0.348 |  | 0.863 |  | 2.828 |  |
| *mompS* | -1.392 |  | -1.649 |  | -1.851 |  | 3.086 |  |
| *p-value<0.05  **p-value<0.01  ***p-value<0.001 | | | | | | | | |
